# Supplementary material for: Cross-cultural adaptation and validation of the Chinese version of the short-form of the Central Sensitization Inventory (CSI-9) in patients with chronic pain: A single-center study
Source: PLoS One. 2023 Mar 16;18(3):e0282419. doi: 10.1371/journal.pone.0282419 (PMC10019621; doi:10.1371/journal.pone.0282419)
Supplement: S1 Table — (DOCX) [file pone.0282419.s002.docx]

**S1 Table. Answers to part B of the Chinese 9-item Central Sensitization Inventory.**

| **No.** | **CSS-related condition (part B)** | Chronic pain population (*n* = 235) | **Healthy population** (*n* = 55) |
| --- | --- | --- | --- |
| 1 | Restless leg syndrome | 4 (1.7%) | 0 (0.0%) |
| 2 | Chronic fatigue syndrome | 2 (0.9%) | 0 (0.0%) |
| 3 | Fibromyalgia | 121 (51.5%) | 0 (0.0%) |
| 4 | Temporomandibular joint disorder | 4 (1.7%) | 0 (0.0%) |
| 5 | Migraine or tension headache | 40 (17.0%) | 0 (0.0%) |
| 6 | Irritable bowel syndrome | 8 (3.4%) | 0 (0.0%) |
| 7 | Multiple chemical sensitivities | 5 (2.1%) | 0 (0.0%) |
| 8 | Neck injury (including whiplash) | 11 (4.7%) | 0 (0.0%) |
| 9 | Anxiety or panic attacks | 49 (20.9%) | 0 (0.0%) |
| 10 | Depression | 30 (12.8%) | 0 (0.0%) |
|  | Average number of diagnoses | 1.17 ± 1.14 | 0.00 ± 0.00 |
|  | 1 central sensitivity syndrome | 90 (38.3%) | 0 (0.0%) |
|  | 2 central sensitivity syndromes | 39 (16.6%) | 0 (0.0%) |
|  | ≥3 central sensitivity syndromes | 31 (13.2%) | 0 (0.0%) |

Data are shown as *n* (%) or mean ± standard deviation.
