# Supplementary material for: Cross-cultural adaptation and validation of the Chinese version of the short-form of the Central Sensitization Inventory (CSI-9) in patients with chronic pain: A single-center study
Source: PLoS One. 2023 Mar 16;18(3):e0282419. doi: 10.1371/journal.pone.0282419 (PMC10019621; doi:10.1371/journal.pone.0282419)
Supplement: S2 Table — (DOCX) [file pone.0282419.s003.docx]

**S2 Table. Answers to part A of the Chinese 9-item Central Sensitization Inventory.**

| No. | **CSI item (part A)** | Chronic pain population  (*n* = 235) | **Healthy population**  (*n* = 55) | ***P*** |
| --- | --- | --- | --- | --- |
| 1 | I feel sleepy after waking in the morning | 2.48 ± 1.19 | 0.96 ± 0.86 | <0.001 |
| 2 | I feel muscle stiffness and pain | 3.05 ± 0.96 | 0.40 ± 0.68 | <0.001 |
| 3 | I feel achy | 2.64 ± 1.24 | 0.16 ± 0.42 | <0.001 |
| 4 | I have a headache | 1.57 ± 1.24 | 0.42 ± 0.60 | <0.001 |
| 5 | I do not sleep well | 2.46 ± 1.30 | 0.76 ± 0.88 | <0.001 |
| 6 | I have trouble concentrating | 1.86 ± 1.22 | 0.45 ± 0.72 | <0.001 |
| 7 | Mental stress makes my body feel worse | 1.81 ± 1.33 | 0.35 ± 0.58 | <0.001 |
| 8 | My neck and shoulder muscles are tense | 2.67 ± 1.23 | 0.76 ± 0.92 | <0.001 |
| 9 | I have a bad memory | 2.44 ± 1.15 | 0.82 ± 0.77 | <0.001 |
|  | Total | 20.96 ± 7.06 | 5.09 ± 3.66 | <0.001 |
