# Supplementary material for: Cross-cultural adaptation and validation of the Chinese version of the short-form of the Central Sensitization Inventory (CSI-9) in patients with chronic pain: A single-center study
Source: PLoS One. 2023 Mar 16;18(3):e0282419. doi: 10.1371/journal.pone.0282419 (PMC10019621; doi:10.1371/journal.pone.0282419)
Supplement: Attachment — Submitted filename: Response to Reviewers.docx [file pone.0282419.s005.docx]

**Manuscript ID:** PONE-D-22-25986

**Title:** Cross-cultural adaptation and validation of the Chinese version of the short-form of the Central Sensitization Inventory (CSI-9) in patients with chronic pain: a single-center study

**Name of Journal**: PLoS One

**Response to Reviewers’ comments**

Dear Editor PhD. Fatih Özden:

Thank you for carefully considering our manuscript! We are grateful for your response and overall positive feedback. After carefully reviewing the comments made by Reviewer 1, we have modified the manuscript, providing a full context to the study.

We hope that you now find the revised manuscript suitable for publication and look forward to contributing to your journal. We believe that the revised manuscript would be of interest to your readership.

Please do not hesitate to contact me in case of any questions or concerns regarding the current manuscript.

Best regards,

Dong-feng Liang

**Reviewer #1**

*Reviewer #1: Most of the issues have been addressed by the authors, except the following:*

*1. In the Abstract (lines 33-35) and Discussion (lines 366-367), it is still stated that “confirmatory factor analysis extracted three main factors (“physical symptoms”, “emotional distress”, and “headache/jaw symptoms”)”. Please clarify and correct this.*
 **Response:** We thank the Reviewer for pointing out that discrepancy. It was corrected.

*2. In Table 4, test-retest reliability, were* *the ICC values shown for each item resulted from the remaining items after deletion of that item? If that is the case, this should be explained in the footnote of the table for clarity. The same explanation should also be applied in Table 6 for the internal consistency.*

**Response:** The Reviewer is right. It was added as a footnote to Tables 4 and 6.

*3. The number of participants for pilot testing of the Chinese CSI-9 is 10 in the Discussion section (line 453) while this is reported to be 6 in the Methods section (line 164). Please clarify and correct this.*

**Response:** We thank the Reviewer for pointing out that discrepancy. It was corrected in the Discussion. It was six.
