# Supplementary material for: Cross-cultural adaptation and validation of the Chinese version of the short-form of the Central Sensitization Inventory (CSI-9) in patients with chronic pain: A single-center study
Source: PLoS One. 2023 Mar 16;18(3):e0282419. doi: 10.1371/journal.pone.0282419 (PMC10019621; doi:10.1371/journal.pone.0282419)
Supplement: Attachment — Submitted filename: Response to Reviewers.docx [file pone.0282419.s006.docx]

**Manuscript ID:** PONE-D-22-25986R2

**Title:** Cross-cultural adaptation and validation of the Chinese version of the short-form of the Central Sensitization Inventory (CSI-9) in patients with chronic pain: a single-center study

**Name of Journal**: PLoS One

**Response to Reviewers’ comments**

Dear Editor PhD. Fatih Özden:

Thank you for carefully considering our manuscript! We are grateful for your response and overall positive feedback. After carefully reviewing the comments made by Reviewer 1, we have modified the manuscript, providing a full context to the study.

We hope that you now find the revised manuscript suitable for publication and look forward to contributing to your journal. We believe that the revised manuscript would be of interest to your readership.

Please do not hesitate to contact me in case of any questions or concerns regarding the current manuscript.

Best regards,

Dong-feng Liang

**Reviewer #1**

*All the concerns have been addressed except the criteria of model fit indices for the confirmatory factor analysis (CFA) and their citation are missing in the Methods - Validity Assessment section. The authors should provide this information to allow readers to evaluate the model fit of the one-factor model from the CFA.*
 **Response:** We thank the Reviewer. It was added in the Methods section.

**Revised version**: The indices used to determine the model fit were: chi-square degrees of freedom (χ^2^/df), goodness-of-fit index (GFI), adjusted goodness-of-fit index (AGFI), comparative fit index (CFI), Tucker-Lewis Coefficient (TLI) and root mean square error of approximation (RMSEA). A model with χ^2^/df<3, RMSEA<0.08, GFI>0.90, CFI>0.90, and TLI>0.90 suggested a good fit [reference].

Reference: Qiu H, Huang C, Liu Q, Jiang L, Xue Y, Wu W, Huang Z, Xu J. Reliability and validity of the Healthy Fitness Measurement Scale Version 1.0 (HFMS V1.0) in Chinese people. BMJ Open. 2021 Dec 7;11(12):e048269.
